# Micro-Hole Drilling on Glass Substrates—A Review

**DOI:** 10.3390/mi8020053

**Published:** 2017-02-13

**Authors:** Lucas A. Hof, Jana Abou Ziki

**Affiliations:** 1 Department of Mechanical & Industrial Engineering, Concordia University, 1455 de Maisonneuve Blvd. West, Montreal, QC H3G 1M8, Canada; l_hof@encs.concordia.ca; 2 Bharti School of Engineering, Laurentian University, Sudbury, ON P3E 2C6, Canada

**Keywords:** micro-drilling techniques, glass, micro-devices, micro-fluidics, MEMS

## Abstract

Glass micromachining is currently becoming essential for the fabrication of micro-devices, including micro- optical-electro-mechanical-systems (MOEMS), miniaturized total analysis systems (μTAS) and microfluidic devices for biosensing. Moreover, glass is radio frequency (RF) transparent, making it an excellent material for sensor and energy transmission devices. Advancements are constantly being made in this field, yet machining smooth through-glass vias (TGVs) with high aspect ratio remains challenging due to poor glass machinability. As TGVs are required for several micro-devices, intensive research is being carried out on numerous glass micromachining technologies. This paper reviews established and emerging technologies for glass micro-hole drilling, describing their principles of operation and characteristics, and their advantages and disadvantages. These technologies are sorted into four machining categories: mechanical, thermal, chemical, and hybrid machining (which combines several machining methods). Achieved features by these methods are summarized in a table and presented in two graphs. We believe that this paper will be a valuable resource for researchers working in the field of glass micromachining as it provides a comprehensive review of the different glass micromachining technologies. It will be a useful guide for advancing these techniques and establishing new hybrid ones, especially since this is the first broad review in this field.

## 1. Introduction

Micromachining is one of the most important aspects among state-of-the-art manufacturing technologies. In the constantly emerging field of micro-electro-mechanical-systems (MEMS) and miniaturized total analysis systems (μTAS), silicon and glass are the primarily used materials.

Many applications need glass because of its unique properties [1,2,3,4,5,6,7,8,9,10]. The micro-optical-electro-mechanical-system (MOEMS) uses glass due to its optical properties, and radio frequency (RF)-MEMS applications take advantage of its good isolation properties [3,4]. Dimensions of the structures to be machined vary from sub-micron to sub-mm and aspect ratios of 0.1 up to 10 or higher. In the packaging process, glass is common as a die for thermal compensation for two of the most commercialized MEMS devices—piezoresistive pressure sensors and accelerometers [3]—and glass can be used as a core material for interposer substrates for laminated semiconductors [4,5]. Typical feature sizes of 50 μm with a depth of 100 μm are required. Glass in this case is chosen due to its good ability to bond to silicon, its similar coefficient of expansion compared to silicon, and its low electrical conductivity [4,5,9]. Bio-MEMS devices are fabricated on glass substrates due to its optical transparency, hydrophilicity, chemical stability and bio-compatibility [3]. The emerging field of Lab-on-a-chip devices for energy (e.g., oil and gas) applications demand high temperatures (subsurface temperatures increase at 30 °C·km^−1^), high pressures (pressure increase at 10 MPa·km^−1^) and volatile fluids, requiring the mechanical strength and chemical properties of glass [10]. Other examples where micromachined glass is used due to its optical transparency and mechanical properties are optical data storage devices and spacers for cellphone cameras.

An important challenge is connecting the micro-components of a microfluidic device to the macro-environment of the world. This is often referred to as the macro-to-micro interface, interconnect, or world-to-chip interface. For commercial success of any microfluidic device, especially for high throughput applications where manual manipulation is not economical, the macro-to-micro interface must be developed [11,12]. Machining of high-aspect ratio micro-holes in glass is the first requirement to manufacture these interconnects. Recently, drilling high density through glass vias (TGVs) became more important for the development of thin (~100 μm) glass interposers in new 2.5D and 3D chip package strategies, due to the demand for higher functionality in small consumer electronics [4,5]. These, often metalized, TGVs (diameters: 10 to 50 μm, depths: up to 100 μm) are used to connect traces and pads on the top and bottom surfaces of the glass interposers [4,5,13,14].

Nowadays, there is a wide variety of methods for glass machining of micro-holes which includes conventional mechanical drilling and non-conventional drilling methods. These methods can be categorized as: (1) mechanical, such as ultrasonic drilling, powder blasting or abrasive jet micromachining (AJM), abrasive slurry jet machining (ASJM) and abrasive water jet machining (AWJM); (2) thermal, such as laser machining; (3) chemical, including wet etching, deep reactive ion etching (DRIE) or plasma etching; and (4) hybrid technologies (which combine two or more methods of the aforementioned for better machining outcomes), such as spark-assisted chemical engraving (SACE), vibration-assisted micromachining, laser-induced plasma micromachining (LIPMM) and water-assisted micromachining. Each method has its advantages and limiting factors on the achievable machined features, including the range of hole diameters, aspect ratios, surface roughness, and machining speed, as well as its associated costs of investment and operation. While review papers exist about machining micro-holes on glass substrates, each review is specific to certain technologies, such as the quantitative comparison of various hole-drilling methods on glass using different laser-machining techniques [15] and review papers on hybrid processes [16,17,18,19] and tool-based micromachining processes [20].

A comprehensive overview of commonly used technologies for machining micro-holes on glass is presented and discussed in this work. Important characteristics of each technique, e.g., achievable aspect ratio, machining speed and machined dimensions, are listed in Table A1 of Appendix A based on the academic and industrial literature. Final conclusions of the different technique capabilities (surface roughness, aspect ratio and machining speed) are presented in a graph, thereby delineating the best-suited machining technique for each application.

## 2. Common Glass Micro-Drilling Techniques

Glass is, by definition, a mixture of oxides, whereby their composition and concentration determine the main properties of the glass. Contrary to fused silica, which is formed of pure SiO_2_, a wide range of other glasses contain different kinds of network modifiers, like boron in the widely used Borosilicate glass. As a result, there are a large number of glass types available on the global market, each with different characteristics and applications.

A glass microfabrication technology is chosen for a certain device depending on the glass type (as each type has a different micro-structure [3,21]) and on the required device properties. The main common challenge for glass micromachining technologies, though, is to deal with the relatively large glass hardness and brittleness. Since conventional techniques such as mechanical drilling have their limitations, a wide range of different non-conventional techniques are used for glass micro-hole drilling. In this review, these methods are discussed by being grouped into mechanical, thermal, chemical, and hybrid drilling processes. Throughout the text, the surface roughness will be described by the arithmetical mean deviation, *R_a_*.

### 2.1. Mechanical Methods

#### 2.1.1. Mechanical Drilling

Mechanical drilling is the most conventional and relatively low-cost method to drill micro-holes in glass. Most often, peck drilling (depth of cut is sub-divided into small drilling cycles [22]) is applied to evacuate chips created inside the holes during drilling [23].

Reported aspect ratios vary from 0.33 to 3.96 with corresponding depths of respectively 130 μm to 4 mm. Typical drilling feed rates are around 5 μm/s [24], which can be increased up to 125 μm/s under special conditions [25].

Mechanical drilling is simple, cost effective and potentially suited for rapid prototyping as it is a mask-less process. However, it may easily result in cracks due to deformation of glass by the thrust force of the drill acting at the bottom surface of the workpiece [23,24,25]. Cracks are more pronounced at the exit of holes than their entrance. Exit cracks on glass plates are mainly cone cracks of relatively large diameter. It is reported in [25] that crack size (typically 50 μm) can be reduced to 15 μm by decreasing thrust forces (from typical values of 2.5 N reduced to 0.8 N), but drilling at low thrust forces makes the process long and impractical. Moreover, using sacrificial pads to support the glass sample to be machined reduces chipping and crack formation [23,24,25]. Changing the cutting conditions may also reduce cracks (down to 29 μm), as shown in [23], when drilling with 0.3 mm cemented carbide micro-drills at a spindle speed of 35,000 rpm and a feed rate of 3 mm/min. Diamond-abrasive drills result in large cone cracks (390 μm), hence radial and median cracks rarely occur [23]. Tool wear is typically much higher for cemented carbide tools compared to diamond tools (>40%). Another approach is to integrate force-feedback (typical forces are around 8 N) in the drilling setup to ensure an optimal feed rate (5–7 μm/s) with minimal chipping [24]. Chipping (typical 70 μm) can be reduced by more than 50% on optical-grade glass.

Research has shown that also exit cracks could be reduced (from 50 μm down to 10 μm) upon attaching a supporting backplate with liquids, including alcohol, water and oil [24,25]. Although several methods to reduce cracks during mechanical drilling are reported [23,24,25], machined surfaces are normally rough. This limits the mechanical drilling applicability to precision micro-device fabrication. In addition, diameters of holes that can be machined are limited to 100 μm [25], and costly high-strength tooling is required to keep the samples in place during machining.

#### 2.1.2. Powder Blasting

Powder blasting—also referred to as abrasive jet machining (AJM), impact abrasive machining or sand blasting—is a technique where a particle jet is directed towards a workpiece for mechanical material removal [7,26,27,28,29,30]. Fine abrasive particles (<100 μm) are propelled by compressed air at the workpiece where material is mechanically removed due to small chipping. Alumina (Al_2_O_3_) particles are commonly used as abrasives [28,30]. To localize material removal, an elastic, particle-resistant foil is placed as mask material. For applications requiring bonding the workpiece to another sample, this mask is added around the hole during blasting to protect the surface from damages caused by the backscattered particles which may jeopardize the bonding process [29]. When using specially designed photoresists as a mask, precise structures (tolerance < 25 μm) can be machined, like in photolithography, in any sort of glass. Adjustment of the angle between nozzle and sample and using multiple nozzles are some options to control and/or decrease the dependence of width and depth of machined features with this technique. Resist-foil masks are typically removed in 10% KOH solution at room temperature followed by ultrasonic cleaning to ensure particle removal from inside the etched structures [30].

Powder blasting is a fast drilling process on brittle materials with no resulting burrs, surface micro-cracks, or heat-affected zone (HAZ) around the machined holes. However, the resulting machined surface is rough (*R_a_* is several microns). This technique is cost effective for relatively large batch sizes as it operates outside a cleanroom environment [30] and can, as it is mask-based, machine holes in parallel. However, this process is not very suitable for rapid prototyping of structures in glass.

Feature sizes down to 30 μm can generally be obtained with aspect ratios up to 2.5. Drilling speeds vary from 0.1 μm/s to 32 μm/s. According to the physics of powder blasting, a taper angle (~15° [2]) is produced for through holes resulting in a narrow hole exit compared to its entrance. This limits the aspect ratio to a maximum of 2.5 [28,29], which can be improved if blasting is performed from both sides of the workpiece; however, this requires precise alignment of the workpiece. Masks and small abrasive particles (<30 μm [28]) are needed for blasting, making the lower limit of the hole diameter around 50 μm. Actual research shows that the mask material affects the hole size. The utilization of higher-resistant mask material, like electroplated copper, can reduce feature sizes from 75 μm down to 50 μm [28]. Moreover, working with smaller ablation particles (~9 μm) further enhances the aspect ratio. During the machining process, particles stick usually to the workpiece surface which leads to difficulties in further fabrication steps like bonding substrates. Post-processing of the powder-blasted workpiece by, for example, wet etching, is a possible solution. Although powder blasting is not clean, it is particularly interesting to different companies (e.g., early recognized by Philips) as it can machine thousands of through holes simultaneously at high accuracy which makes it a well-established technology in micro-manufacturing.

Techniques similar to abrasive jet machining have been reported [31,32,33]. These methods include abrasive slurry jet micromachining (ASJM) and abrasive water jet micromachining (AWJM) which make use of abrasive slurries or water to machine blind and through holes in glass. Reported values for machined diameters vary from 390 μm to 2 mm with aspect ratios of respectively 0.9 and 1.5 machined at corresponding feed rates of 4.4 μm/s and 0.6 μm/s.

These techniques are discussed below:

(A) Abrasive slurry jet micromachining (ASJM)

In ASJM, a slurry with abrasive particles (typically 1 wt % 10 μm Al_2_O_3_ particles [33]) is pumped through a small orifice (~180 μm) and the derived jet is directed to the workpiece causing material removal. ASJM operates normally at pressures of 1 MPa to 14 MPa [32]. Although this technique does not require mask materials, research investigations show the possibility to reduce frosted areas around the holes when using sacrificial polymeric or glass surfaces [33].

Features of this technology are its machining flexibility, absence of HAZ around the holes and the non-pronounced tool wear. However, the resulting holes have frosted areas at the entrance and the inner walls are not flat. Additional process steps can be applied to overcome frosted areas such as using different slurry additives, e.g., polyethylene oxide (PEO) [33].

(B) Abrasive water jet micromachining (AWJM)

Although the machining mechanism in AWJM shows many similarities with the ASJM technology, the main difference is the high-pressure operation of AWJM (up to 345 MPa [33]) compared to low-pressure ASJM (typically around 1–14 MPa, although up to 70 MPa is reported [32]). Similar to ASJM, tool wear is not measurable and no HAZ are present. However, chipping occurs at the exit of the through holes, which is most likely due to the high operating pressure [33].

#### 2.1.3. Ultrasonic Drilling

This abrasive process comprises a vibrated tool, a slurry supply unit, and a movable machine body to which the workpiece is mounted. During ultrasonic machining (USM), the tool (called sonotrode) oscillates at high ultrasonic frequencies, usually 20–40 kHz with an oscillating amplitude of several microns, and hammers abrasive particles (e.g., boron carbide (B4C) grits with a size of 5 μm) into the hard-brittle workpiece [34]. This causes indentation, micro-cracks and finally material removal. When reducing tool diameter, abrasive grain size and vibration amplitude to the micro-scale, this technology is referred to as micro ultrasonic-assisted lapping [35]. For this technique, several innovative strategies are applied to the machine, such as helical tool rotation (50 rpm), on-machine tool preparation by electrical discharge machining (EDM) to avoid alignment issues, workpiece vibration, and force feedback control loops [35,36,37,38,39]. Micro-ultrasonic-assisted lapping can produce very small diameter holes (down to 10 μm) with straight sidewalls [35,40] and high aspect ratios (up to 10). The tool wear is high, however, making redressing operations necessary for every 25 to 50 machined holes to avoid feature degradation. USM requires rather large capital investment and operates at relatively low feed rates. Moreover, the machined surface presents sometimes chipping and cracks in the subsurface. Average surface roughness is typically >10 μm, while it can be improved down to 1 μm *R_a_* when using micro-pins for grinding operation [41,42].

Minimum hole diameters obtained by ultrasonic machining are typically around 150 μm with aspect ratio 4 and drilled at feed rate 0.15 μm/s; however, the lowest USM diameter on glass is reported as 10 μm [41]. Here, EDM-machined cemented tungsten carbide micro pins were deployed. As well, feed rates of 16.67 μm/s can be achieved when using diamond core drills and machining relatively large holes (~950 μm).

### 2.2. Thermal Methods

#### 2.2.1. Laser Machining

Material removal by thermal shock or ablation can be achieved by laser-based processes. This may be used to drill micro-holes in glass. Transferring photon energy of the laser light to glass is challenging, however, as the last is transparent to a wide range of wavelengths [43,44], which requires generation of high peak intensities to trigger a nonlinear absorption effect. Carbon dioxide (CO_2_) lasers are among the most frequently used lasers for industrial applications over long periods, since its equipment is relatively simple and requires low capital investment [45]. At present, different laser processes resulting in innovative hybrid technologies, are being developed by many research groups and industries like Femtoprint [46] and Fraunhofer ILT [47]. These two use ultra-short pulse (USP) laser as a preprocessing ‘flexible masking method’ and wet etching as a second step to obtain the desired structure. (The laser-treated areas have enhanced etch rates (20–50 times higher than untreated surfaces) and therefore etching is favored in these areas (i.e., acting like a mask to define the structure geometry)).

Despite all extensive research and development, laser systems still suffer from HAZ, ranging from sub-micron (USP-laser) dimensions to tens of microns (CO_2_-laser), and bulges around the rims of the machined holes (typical height: 15 μm) caused by recast (debris). This causes difficulty to bond the glass substrate after machining. Hole diameters, machined with liquid-assisted femtosecond lasers down to 5 μm, with aspect ratios as high as 70, have been reported [48]. There is no upper limit in achievable hole sizes and a typical roughness value, *R_a_*, is around 1 μm. Machining speed per hole differs from 30 μm/s [48] up to 2000 μm/s [13] depending on laser type and desired quality.

Novel strategies like PDMS masking [49] and using ultra-short pulse lasers (femtosecond pulses) [48,50,51,52,53,54,55,56,57], already succeeded in reducing the unwanted side effects of laser machining. Bulge heights around the hole entrances can be reduced by factor 13 to 1.2 μm using 150 μm-thick PDMS masks and a 10–15 W CO_2_ laser [49]. Furthermore, preheating the workpiece proved to reduce thermal stresses by reducing the temperature gradient [49,51,54,58]. Improved aspect ratios of micro-holes can be achieved using two laser beams on opposite glass surfaces [45,59].

Another option besides CO_2_ lasers and ultra-short pulse lasers is the nanosecond-pulse, Q-switched diode-pumped solid state (DPSS) laser, which is a good trade-off in terms of technological complexity, costs and quality [45,60]. Pulse energies around 200 μJ and 100 kHz repetition rates were reported for machining 5 mm holes in Gorilla glass^®^ [61] with DPSS lasers.

In general, laser systems are flexible. Most do not need masking layers as they are direct-write technologies, but they are still expensive. The high throughput of laser machining of glass makes it a good option for the MEMS industry, wherein large amounts of holes have to be produced. The most popular laser-drilling types are summarized in the following:

(A) Carbon dioxide (CO_2_) laser

The CO_2_ laser technique is a serial thermal laser process which removes material through ablation by relatively long pulses. This causes a thermal impact on the glass and generates mechanical stress, which leads to crack formation during cooling. Many solutions are investigated to reduce this phenomenon, like local preheating of the workpiece, heating of the entire workpiece during drilling and thermal post-treatment of the drilled substrate using an oven [13,25,51,62]. In fact, smooth surfaces (*R_a_* ~ several microns) are possible to achieve due to the generated heat [63]. Hole diameters on glass down to 25 μm with aspect ratio 4 and machined at 20,000 μm/s per hole are claimed [64]. Although the reliability of CO_2_ laser drilling of glass is low, its fast drilling speed and low equipment costs make it a good option for industry. Some examples of CO_2_-laser-machined micro-holes in 500 μm-thick glass (Schott D263Teco, SCHOTT AG, Mainz, Germany) are illustrated in [63]. These holes have relatively high aspect ratio and high conicity.

(B) Excimer laser

The excimer lasers are gas-type lasers that offer access to the ultraviolet (UV) or deep UV region with short pulse rates and durations (respectively 1–100 Hz and 5–50 ns). This results in high pulse intensity and high resolution, making excimer lasers suitable for machining glass materials where high precision and good surface quality are required. While CO_2_ and solid-state Nd:YAG lasers are generally employed in direct writing (serial mode) during machining, excimer lasers are normally used for projection printing (parallel mode), which has higher throughput [6]. Some typical excimer-laser-drilled micro-holes machined at 500 Hz repetition rate and energy levels 4–5 J/cm^2^ show bulges around the rims on the hole entrance (bulge heights around 10 μm) [59]. However, when using lower laser fluence, reduced cracks and material break-off results. Drilling from both sides can also eliminate these problems while enlarging the diameter at the rear side of the workpiece, which lowers the taper angle [59,65]. Reported hole diameters range from 30 μm to 200 μm [59] with aspect ratios of 2.2 [53] up to 16.7 [65].

(C) Liquid-assisted laser processing (LALP)

Liquid-assisted laser processing (LALP) was developed to reduce the formation of bulges on the rims of machined holes and residual stress reduction [62]. Machining is done while the substrate is immersed in water to reduce the temperature gradient, bulges and HAZ region. Chung et al. [62] deployed a 6 W CO_2_ laser and they quantified as well the reduction in efficient laser power in LALP machining, e.g., at four passes and constant initial laser power (6 W), the machined depth decreased by 100 μm upon 0.5 mm water depth. The residual stress is reduced by 136 MPa when the sample is immersed in 1 mm water and a 100 μm hole is machined.

The bulges are mainly caused from re-solidification of evaporated debris. LALP reduces the bulge height by the stronger natural convection in water, due to the laser heating, which carries the debris away [51,62].

This technology is attractive for improved CO_2_ laser machining, saving the costs of moving to technologically complex and expensive methods (e.g., USP laser). Machined holes of 280 μm are reported with aspect ratio ~2 μm at a speed of 11,400 μm/s.

(D) Polydimethylsiloxane (PDMS) protection mask

Upon, protecting the glass workpiece with a polydimethylsiloxane (PDMS) layer, the temperature gradient in laser machining is reduced. This decreases HAZ formation and can result in crack-free machining of Pyrex glass [49]. The PDMS protection layer also eliminates common defects and diminishes the bulge height around the hole entrance by a factor of 13 compared to the process in air (without PDMS cover layer) to 1.2 μm. Moreover, the feature sizes that can be machined are reduced by 10%. CO_2_ laser machining in combination with 150 μm-thick PDMS masks are used by Chung et al. [49] reporting hole diameters of 120 μm with aspect ratio 4.

(E) Ultra-short pulse (pico/femtosecond) laser

Ultra-short laser pulses do not produce a large HAZ due to the smaller amount of heat penetration into the glass sample [52,53]. These lasers can induce strong absorption even in materials that are transparent to the laser wavelength. This method can produce smooth holes with small diameters (7–10 μm) and depths of 30 μm in fused silica, without forming micro-cracks or surface welling [52]. However, this high quality can only be obtained at reduced process speed (~30 μm/s [48]). For example, excimer lasers with nanosecond pulse width are still much faster (typically 10 times faster) [53,59]. As for the equipment costs, they are relatively high compared to other laser techniques such as CO_2_ lasers.

(F) Laser-induced plasma

To machine small-sized shallow features with very smooth surface finish (*R_a_* = 50 nm), laser-induced plasma can be used. The key to this method is the production of charged particles by targeting the focused laser beam on a metal surface [50].

Spherical crater-like blind holes with a typical diameter of 15 μm and a depth of 4.5 μm are formed. This technique cannot machine high aspect ratio through holes.

(G) UV laser with absorbent powder

To machine high aspect ratio micro-holes with reduced micro-cracks, research is conducted with a nanosecond pulsed laser and absorbent powder. This powder is deposited on the glass surface and on the bottom of the machined holes. The deposition is repeated during machining. Although fewer cracks are formed in this case, several micro-cracks are present, as witnessed by the non-transparency of the hole [58]. Aspect ratios of 12 and higher and hole diameters of 200 μm at 100 μm/s are achieved by Kono et al. [58].

#### 2.2.2. Focused Electrical Discharge Method

Recently, a through-glass via (TGV) formation method by electrical discharging was introduced: focused electrical discharge method [4,5]. This technology, where the targeted glass is kept in a space between two axial aligned electrodes, consists mainly of two steps. First, the electrical discharging is focused and controlled to generate heat, which decreases the glass viscosity locally. Second, dielectric breakdown and internal high pressure occurs due to Joule heating. This results in the ejection of glass. This process can produce small diameter holes (down to 20 μm [4]) precisely in thin glass workpieces (100 μm to 500 μm) during a relatively short time (200 ms to 500 ms). Aspect ratios of 5 up to 7.6 and machining speeds of 200–500 μm/s are achieved by Takahashi et al. [4]. Annealing is needed in order to remove the residual stresses. High aspect ratio and smooth-machined surfaces are obtained. Fabricating ultra-thin glass interposers in laminated semiconductors is the main targeted application of this method [5].

### 2.3. Chemical Methods

#### 2.3.1. Wet Etching

Glass machining by wet etching is due to dissolving glass by immersing the workpiece in an etchant, most commonly hydrofluoric acid (HF). Mask material, which must be etchant resistant, is used to define the pattern to be removed [1,66,67]. When applying intermediate masks, multiple levels can be machined using this process. Due to the amorphous nature of glass, the process is isotropic, resulting in rounded sidewalls and undercutting and low aspect ratio machining (<1). Pinholes and notching defects on the edges of etched structures are other limitations of this process. These defects are mainly due to the residual stress in the mask, the compressive or tensile stress, the stress gradients (for multilayer mask), and the hydrophobicity of the masking surface [66]. Partial improvements can be achieved when optimizing the mask material, i.e., enhancing etchant resistance, and annealing of the workpiece. Although this results in higher etch rates, it causes higher surface roughness. Small, highly detailed features (hole diameters greater than 1 μm [68]) with smooth surfaces (30 nm to 60 nm *R_a_* [26]) and aspect ratio <1 can be created with wet etching by using accurate lithography-fabricated masks. Roughness and etching rate are strongly influenced by the glass composition. The presence of some oxides (such as CaO, MgO, Al_2_O_3_) in the glass composition give insoluble products in HF solution [69]. A large number of holes can be machined at the same time, as the technique is a batch process. Typical etching speeds vary from 0.07 μm/s to 0.24 μm/s. No micro-cracks and no HAZ are formed around the features [3,66]. Wet etching with highly concentrated HF (around 50%) is, however, hazardous to the environment and humans as it uses an acid etchant—even low concentrations (>2%) are already seriously toxic.

Recently, a novel wet-etching technology, electrochemical local acidification of fluoride-containing solution, was introduced [70]. The central idea is to produce the highly toxic hydrofluoric acid (HF) locally near a tool electrode where this causes local etching of the glass substrate around the tool tip. Using this method, holes can be machined at a slightly higher speed (0.45 μm/s) than standard HF etching, and no masks are required [70]. Systematic study will be necessary to optimize this technology for specific applications.

#### 2.3.2. Deep Reactive Ion Etching (DRIE)

Deep reactive ion etching (DRIE), or deep plasma etching, relies on sulfur hexafluoride [71,72], perfluorocyclobutane [73] or trifluoromethane [74] gases as the main etch precursors (dissociated into radicals and ions) for both chemical and physical etching, as in plasma etching. Although the gas chemistry is geared more towards silicon etching, glass can be processed as well [2]. In glass, the fluorine radicals carry away the silicate, and carbon difluoride radicals carry away the oxygen as volatile compounds. To direct the ions and create the desired features, metal masks can be used such as nickel with a gold-chromium seed layer. Other studies investigated the use of silicon wafers, a-silicon, and SU-8 as mask material [71,73,75,76]. DRIE can compete with other glass deep-etching technologies in terms of aspect ratio, wall verticality, feature depth and throughput. Very small, accurate features (diameters down to 1 μm) with smooth surfaces (*R_a_* = 2 nm [77]) and high aspect ratio (up to 40 [77]) can be achieved in this highly anisotropic process.

The major disadvantages of DRIE are the amount of process steps needed (e.g., different masks), and the extremely low etch rate (around 0.009 μm/s), although the number of holes that can be produced simultaneously is greater than 200,000 [9]. Moreover, the process is limited by the relatively low heat transfer of glass (typical thermal conductivity of glass is 100 times lower than silicon) making it challenging to achieve deep-etching and high etch rates.

### 2.4. Hybrid Methods

In order to overcome the limitations encountered while using the above-listed technologies, researchers worked on combining different machining processes, leading to what is called hybrid machining. Many definitions were proposed for hybrid machining, the most common being that hybrid machining is a method by which two or more machining processes are applied independently or simultaneously on a single machine. Recently, hybrid machining was defined by the College International Pour la Recherche en Productique (CIRP) as a process that uses simultaneous and controlled interaction of several machining mechanisms, tools and energy sources to enhance the machining performance [19]. Based on this definition, Chavoshi et al. [16] classified hybrid micromachining processes into two groups: assisted and combined hybrid micromachining processes.

In assisted hybrid micromachining, the major machining process is applied while input from other types of energy is added [78,79]. In combined hybrid micromachining, all the combined micromachining processes simultaneously contribute to the material removal and machining properties. In this category, research is focused on electrochemical processes for machining nonconductive materials while improving the material removal rate and the machined surface quality and reducing the machining time.

The major assisted hybrid glass micromachining techniques and combined hybrid micromachining processes are discussed below.

#### 2.4.1. Assisted Hybrid Micromachining Techniques

(A) Vibration-assisted micromachining

In this process, mainly tool vibration (also sometimes workpiece or machining fluid vibrations) is added to the main machining process. This has been applied to several processes including micro-milling and micro-electrochemical discharge machining (ECDM) [16]. For appropriate combinations of cutting velocity, and vibration amplitude and frequency, the tool periodically loses contact with the chip, resulting in reducing the machining forces and enhancing the tool life and surface finish [18]. Furthermore, higher depth of cut, smoother surfaces, and near-zero burr are achieved compared to conventional machining [80,81,82]. On the other hand, this technique may result in surface cracks due to the hammering of the tool [78].

(B) Laser-assisted micro-cutting/milling

This technique enhances machining of especially hard brittle materials as the laser beam softens the materials to be machined. It is used to machine ceramics and glass where the local softening of the material during the process enables geometrically defined cutting edge, uniform surfaces and reduced surface roughness [19]. For further improvement of surface quality and machining accuracy, these processes can be combined with other ones.

(C) Laser-induced plasma micromachining (LIPMM)

In this method, plasma is induced in a liquid at the focal point of the laser beam which allows micromachining of shiny materials and transparent materials with high reflectivity like glass [83]. The shape of the plasma can be optically or magnetically manipulated to obtain specific micro-patterns while reducing machining time.

(D) Water-assisted micromachining

Machining by laser produces debris which reduces the machined surface quality. To remove this debris while machining, water is added on top of the substrate, resulting in a better machined surface (less taper and heat affected zones) and in an accelerated ablation rate (twice as fast as the case of laser machining in air) [16]. With this technique, high aspect ratio holes could be ablated in silicon, LCD glass and alumina by water-assisted femtosecond and CO_2_ laser pulse ablation. However, due to the rapid solidification of the molten material, rough surfaces result [84].

(E) Chemical-assisted micromachining

In this technique, methanol is added on the substrate surface that is to be machined with laser. Methanol has better wettability and lower boiling temperature than water which enhances cooling and cleaning of ablated particles produced during laser machining. The result is cleaner and smoother surfaces [16].

(F) Chemical-assisted ultrasonic machining (CUSM)

In order to improve the efficiency of ultrasonic machining of glass, hydrofluoric (HF) acid is added to the abrasive slurry but in low concentrations, normally less than 5% HF solution [85]. This leads to increasing the material removal rate for micro-drilling by up to 40% and enhancing the surface quality as HF acid weakens the Si bonds. However, the hole gets enlarged.

(G) Electrorheological (ER) fluid-assisted ultrasonic machining 

In micro-ultrasonic machining of hard and brittle materials like glass, chipping and low machining accuracy are generally the result. To reduce these problems, electrorheological (ER) fluid-assisted ultrasonic machining is used. In this method, electrorheological (ER) fluid is mixed with the abrasive particles and added into the machining zone. This fluid has dielectric particles where increased electric field intensity results in increasing viscosity.

As a voltage is applied between the cathode located on the workpiece surface and the vibrating micro-tool which is the anode, machining results. The resulting electric field in the machining zone in the vicinity of the tool tip increases the ER fluid viscosity and thus traps the abrasive particles (in the ER fluid) beside the tool tip. This results in enhanced machining accuracy and efficiency [86,87,88].

(H) Electrical discharge machining (EDM) with an assisted electrode

Another machining method for glass, which can be used for micro-hole drilling, is micro-electrical discharge machining (EDM) with an assisted electrode [89,90]. The EDM process is based on ablation of material through melting and evaporation, by electrical discharges. These discharges take place upon applying a voltage between the tool electrode and the electrical conductive workpiece, which are separated by a dielectric medium. To achieve machinability of non-conductive materials such as glass with micro EDM, the process has to be initially started by a conductive starting layer on top of the workpiece [89,90]. While machining the starting layer, the dielectric (typically a hydrocarbon oil) is cracked, providing conductive carbon that settles onto the glass surface, generating a new conductive layer that enables the next discharges to take place. This sequence of removing the layer including the underlying targeted material and creating new thin conductive layers can be repeated by controlling the process environment. Non-conductive ceramics could be machined with aspect ratios >5 as reported by Schubert et al. [89]. This process can be used both for serial prototyping using a single tool and it can be used for batch-based manufacturing when using multi-tool heads to produce many holes at the same time. However, preprocessing is needed for deposition of the conductive starting layer, and sophisticated process control is required for stable operation.

(I) Hot embossing

Micro-structuring of glass can also be done by forming processes such as hot embossing which is based on viscous flow of glass at high temperatures. This technology makes use of a micro-patterned mould and a heated glass workpiece and it is mostly used for large batch size fabrication of optical lenses [91]. Almost any possible shape that can be patterned on the metal mould can be transferred to the glass workpiece. A critical parameter is the process temperature. If the temperature is high, this will reduce the glass viscosity, resulting in adherence of the glass to the mould surface. However, if the process is carried out at lower temperatures, glass would have relatively higher viscosity, and will require higher mechanical forces to pattern it. To overcome these issues, the mould surface or glass substrate can be coated to prevent the glass from sticking to the mould [91]. This technology is most suited for large batch size production of features in glass, due to the need of mould fabrication and the setup required for this process, e.g., sophisticated heat control.

#### 2.4.2. Combined Hybrid Micromachining Processes

Micro-electrochemical discharge machining (ECDM) or spark-assisted chemical engraving (SACE).

In this process, used to machine non-conductive materials, a voltage is applied between the tool-electrode (positioned above the substrate) and counter electrode which are both dipped in an alkaline solution. At voltages higher than the critical voltage (around 30 V), bubbles around the tool coalesce into a gas film and discharges are generated through it. Glass machining is possible due to thermally promoted etching and bombardment of discharges [8,92]. Although the performance of this process depends on several parameters including the tool shape and motion, voltage, electrolyte, and machining gap, the machining voltage proved to have a more significant effect on the material removal rate [93,94].

SACE allows manufacturing of small and large holes (up to 2000 μm in diameter) and can produce high aspect ratios (>10), while achieving relatively transparent and smooth machined surfaces (*R_a_* = 0.13 μm) on glass [92,95,96,97]. Compared to laser processes, HAZ are less apparent in SACE, due to the reduced machining temperature (typically ~500 °C compared to ~2000 °C for laser). Also, compared to ultrasonic drilling, wet- and dry-etching of the machining speed per hole is high. However, the surface roughness is higher than that in most conventional wet and dry etch techniques.

Significant research work has been carried out to reduce HAZ and surface roughness by machining at the lowest possible temperature though reducing the critical voltage [98,99], or using pulsed voltage [95,100,101]. Further improvement was achieved by post-processing of machined holes with electrophoretic deposition grinding (EPDG) which results in reduced HAZ, smooth surface and excellent taper angles (as low as 0.2 degrees) [92], while increasing the machining time (by 5 times).

A major problem encountered with SACE is the limited flushing of the machined material at high machined depths which reduces both the machining speed and quality. Several methods were proposed to allow more localized flushing of the machining zone, including:

- Adjusting the tool shape: different tool shapes including tools with side insulation, flat sidewalls, and spherical ends proved to reduce the taper and overcut [102], enhance machining accuracy [103,104,105], and reduce the hole entrance diameter by up to 65% and the machining time by up to 83% for a 500 mm deep hole [106].
- Tool rotation: results in smooth sidewalls (*R_a_* down to 0.13 μm [95]) and reduced taper [96].
- Tool, electrolyte or workpiece vibrations: low frequency vibrations (0–30 Hz) of a cylindrical 400 μm tool increase the material removal rate (MRR) by factor of two [107] where square waveform showed better improved compared to sinusoidal tool vibration [108,109]. Electrolyte ultrasonic vibration (1.7 MHz) shows improvements in machining depth (320 μm to 550 μm), and reduction in taper and overcut when applying ultrasonic vibrations to the electrolyte [110].
- Pulsed voltage: results in better machining and surface finish [111].
- Inducing a local magnetic field: locally stirs the electrolyte which enhances the surface quality and machining depth while reducing machining time (by around 57.4%) and the overcut (by 23.8%) and at low electrolyte concentration [112].
- Using force feedback control algorithms for drilling: algorithms applied to control the tool motion during drilling are promising for improving the machining quality and speed [113,114,115,116].

## 3. Discussion

**Figure 1:**
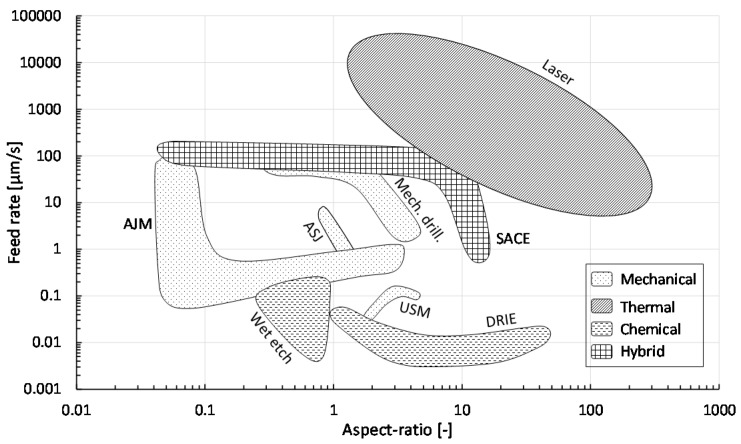
Feed rate (μm/s) vs. aspect ratio (µm/µm) for different glass drilling methods, grouped into four categories (mechanical, thermal, chemical and hybrid). Values in graph based on Table A1.

**Figure 2:**
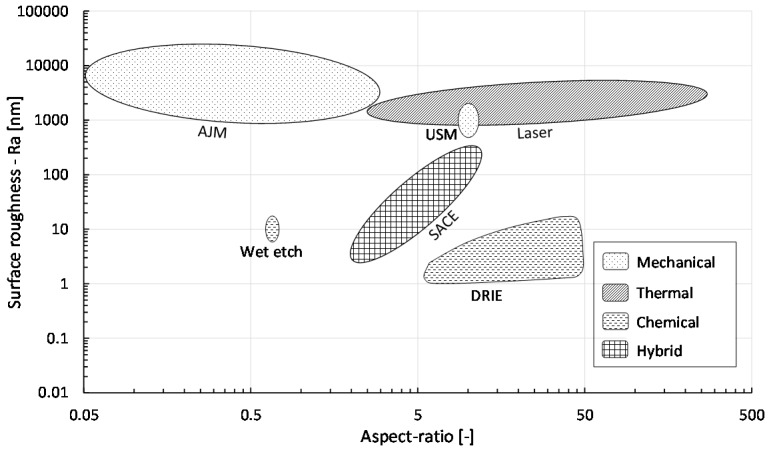
Surface roughness, *R_a_* (nm) vs. aspect ratio (µm/µm) for different glass drilling methods, grouped into four categories (mechanical, thermal, chemical and hybrid). Values in graph based on Table A1.

**Figure 3:**
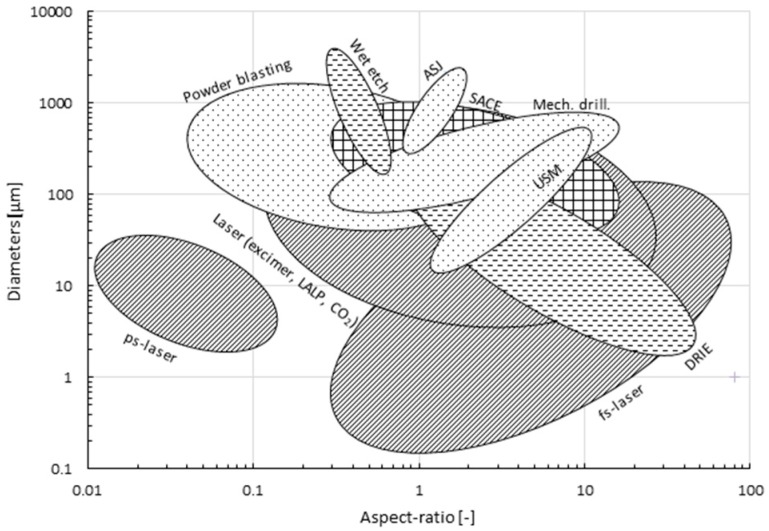
Machined diameters (μm) vs. aspect ratio (µm/µm) for different glass drilling methods, grouped into four categories (mechanical, thermal, chemical and hybrid). Values in graph based on Table A1.

According to [1,11,12], high aspect ratio (>5) and low surface roughness (<1 μm), i.e., smooth surface morphology, of drilled micro-holes are the main requirements to achieve novel glass micro-devices, such as those in MEMS, MOEMS and bio-MEMS. Another important issue, especially for industry, is the machining speed, or more generally the cycle time, since this determines the process throughput and therefore its costs. Targeted hole diameters depend entirely on the application, varying from sub-micron (e.g., many MEMS applications) to sub-millimeter (e.g., smartphone cover glass). A comparison of these outcomes for all described technologies is shown in Figure 1, Figure 2 and Figure 3, constructed based on the values reported in Table A1 (Appendix A). Most of these values were given by the literature as discussed before; however, to have a sufficiently large sample number, more data was added in Table A1 from additional literature [117,118,119,120,121,122,123,124,125,126,127,128,129,130,131]. Each area in the figures represents a different drilling technique. Figure 3 presents the minimum feature size of micro-holes to be machined on glass by the different technologies.

To produce high aspect ratio holes (up to 40) with low surface roughness (*R_a_* ~ 1 μm), a chemical drilling technology like DRIE (Figure 2) can be used. DRIE has further the important ability to machine in parallel a large amount of holes (>200,000) with high accuracy. However, this batch process is slow (etching rates ~ 0.01 μm/s), complex (e.g., masks, operating conditions) and expensive. It has extremely low etch rate (per etched hole), uses sophisticated metal masks, and the equipment is rather expensive and complicated thus limiting its usage in industry. Furthermore, problems may occur when dry etching glasses containing lead or sodium (such as the most commercial standard soda-lime glasses) since this produces non-volatile halogen compounds as reaction products. Exclusively fused silica (formed mainly of silica) can be etched by this process, which restricts its use for a wide range of applications.

Thermal-based technologies, especially laser drilling, also produce high aspect ratio micro-holes (typical ~ 10–50) but with much higher speeds (up to 2000–20,000 μm/s) and less complexity compared to chemical machining (Figure 1). However, a good surface finish is not achievable in this case (*R_a_* > 500 nm) and bulges form around the rims of the hole entrance for glass substrates, which prevent bonding. Similar to chemical processes, thermal processes are also expensive.

Regarding the ease of handling the machining process, mechanical drilling (Figure 1 and Figure 2) is in general the most favorable as it is well-established (e.g., powder blasting). Moreover, it is significantly cheaper than thermal and chemical processes. However, mechanical drilling cannot machine high aspect ratio micro-holes (typical ~ 4) and the resulting surfaces are rough (chipping > 10 μm), requiring costly and time-intensive post-processes (polishing). As shown in Figure 1, Figure 2 and Figure 3, hybrid technologies like SACE provide a trade-off between acceptable machining speed and surface roughness with reasonably high aspect ratio (up to 11) and workable minimum dimensions for most glass applications.

While the above-mentioned comparison presents average ranges and values of hole specifications established in the four machining categories (mechanical, thermal, chemical and hybrid), in all manufacturing cases, specific requirements are needed and trade-offs are always necessary. Figure 1, Figure 2 and Figure 3 allow choosing the machining process based on trade-offs between the aspect ratio and machining quality and speed depending on the fabrication requirements.

For example, for an aspect ratio of 1, chemical (wet etching, ASJ) mechanical (powder blasting, mechanical drilling), thermal (laser drilling) and hybrid (SACE) machining can be used. Wet-etching (chemical) provides the best quality (10 nm *R_a_*) but is the slowest (speed around 0.3 μm/s). A similar speed (0.25 μm/s) can be achieved with powder blasting (mechanical), but the surface roughness can increase from 2500 nm to 10,000 nm *R_a_*. For fastest drilling, laser (thermal) can be used (speed can reach 20,000 μm/s) but quality is not the best (2000 nm *R_a_*). For acceptable speed and quality, SACE drilling (hybrid) can be applied as speed reaches 120 μm/s, which is faster than chemical and mechanical techniques, and the resulting surface is smooth (<200 nm), which has lower roughness than achieved surface roughness by thermal and mechanical processes.

For a high aspect ratio of 10, DRIE etching (chemical) provides the best quality (4 nm *R_a_*) but is very slow (0.01 μm/s speed). Laser machining (thermal) is the fastest (120 μm/s), but roughness is in average around 1000 nm. SACE (hybrid) provides a trade-off between good quality (200 nm *R_a_*) and acceptable speed (reaches 10 μm/s in this case).

In summary, based on these comparisons, wet etching is relatively expensive due to the need of a cleanroom and multiple process steps (e.g., masking), although it is still a good option for mass production of low aspect ratio structures requiring high surface quality. Laser drilling is also relatively expensive, due to the need of sophisticated setups and laser sources, but it is a good option for fast and flexible drilling requiring good surface quality. Mechanical drilling and hybrid technologies such as SACE/ECDM may be the most suitable for prototyping as they are the cheapest processes; unfortunately, they either do not result in good surface quality or are relatively slow.

We constructed Table 1 based on the information presented in this review. Table 1 summarizes the qualitative comparison of the different technologies based on the achievable aspect ratios, machining speed and surface roughness. Low aspect ratios are defined as below 10, low machining speeds refer to speeds below 100 μm/s and low surface roughness (high quality) refers to roughness lower than 100 nm (*R_a_* < 100 nm).

## 4. Conclusions

An overview of commonly used technologies for micro-hole drilling in glass is presented. The technologies are divided into four categories: mechanical, thermal, chemical and hybrid drilling technologies. Based on the review, graphs are constructed for aspect ratio versus machining speed and aspect ratio versus surface roughness to get a comprehensive comparison of the different technologies. Furthermore, a qualitative comparison of the main characteristics of the technologies is summarized in a table. This paper helps in identifying the glass micromachining technology that is currently most suitable for a certain application based on machining requirements.

Each of the drilling technologies has certain limitations. While thermal processes such as laser drilling are fast and flexible, they lack high surface quality. Chemical processes such as wet etching establish smooth surfaces; however, masks are required, resulting in more complexity, low flexibility and higher cost of the process. Mechanical methods such as conventional drilling are relatively slow and exhibit poor surface roughness. To overcome the burdens of certain technologies while taking advantage of the good process outcomes, research is ongoing on developing and implementing hybrid micro-technologies which combine two or more machining technologies to reach an outcome that satisfies most requirements for the desired micro-holes in glass.

## Figures and Tables

**Table 1:** Features of the four main groups of drilling technologies for glass.

Process	Mechanical				Thermal		Chemical		Hybrid
	Mechanical Drilling	Powder Blasting	ASJ	USM	Laser Drilling	FEDM	Wet Etching	DRIE	SACE
Aspect Ratio ^1^	−	−−	−−	−	++	−	−−	++	+
Machining Speed (Serial) ^1^	+	−	−−	−−	++	+	−−	−−	+
Surface Roughness ^2^ (*R_a_*)	−	−−		−	−	+	+	++	+
Minimum Dimensions (μm)	150	50	300	200 (10)	5	20	1	0.5	100
Rapid Prototyping (Serial Mode) ^3^	++	−−	+	+	++	−	−−	−−	++
Mass Fabrication (Parallel Mode) ^3^	−−	++	−	−	−	+	++	++	−−
Tooling Complexity/Costs ^4^	−−	−−	−	−	++	+	−−	−−	++
Applicable to Wide Range of Glass Types ^3^	++	++	++	++	+	−−	−	−−	++
Equipment Costs/Complexity ^5^	++	+	+/−	−	−	−	−	−−	+

On a scale of 1 to 4, the above symbols indicate: (−−) Level 1; (−) Level 2; (+) Level 3; (++) Level 4. Level 1 and Level 4 are indicated for each column on the table. ^1^ low −−, high ++; ^2^ high −−, low ++; ^3^ non-applicable −−, applicable ++; ^4^ complex −−, simple ++; ^5^ expensive −−, cheap ++.

## Appendix A

**Table A1:** List of different drilling techniques with their main feature characteristics for drilling micro-holes in glass.

**Mechanical Methods**	**Material**	**Diameter (μm)**	**Aspect Ratio**	**Taper Angle * (°)**	**Speed (μm/s)**	**Depth (μm)**	**Surface Roughness (nm)**	**References**
Grinding-drilling	optical grade glass and quartz	1011–1323	3.96–3.02	-	5	4000	-	[24]
Micro-drilling	soda-lime glass	100–400	1.3–0.33	-	125	130	-	[25]
Mechanical drilling	glass	>150	4–14	-	slow	-	-	[113]
Powder blasting (30 μm particles)	glass	150–1000	0.07–0.24	-	0.083–0.133	10–240	-	[114]
Powder blasting	glass	<50	2.5	-	0.4	-	2500	[28]
AJM (abrasive jet micromachining)	borosilicate glass	800	<0.06	-	32	50	high	[7]
ASJ (Abrasive slurry jet)	-	390	0.9	-	4.38	350	-	[32]
ASJM (Abrasive slurry jet—Al_2_O_3_ 10 μm particle slurry flowrate:1.67 mL/s)	Borosilicate glass	800	1.13	-	1.88	900	frosting	[33]
	-	2000	1.5	34	0.56	3000	-	[33]
Micro-ultra-sonic (abrasive grains)	pyrex 7740	420	>10	0.6	-	5000	1000	[42]
Ultrasonic (combined with EDM)	glass	150	3–4	-	0.13–0.15	-	-	[115]
Ultrasonic vibration drilling	-	10	2	-	0.05	20	no cracks	[40]
Ultrasonic vibration drilling (combining ultrasonic and low-frequency/diamond core drill)	glass	964	-	-	16.67	-	-	[37]
Ultrasonic grinding (cemented tungsten carbide micro pins)	crown glass	10–30	-	-	0.25–0.27	-	-	[41]
**Chemical Methods**	**Material**	**Diameter (μm)**	**Aspect Ratio**	**Taper Angle * (°)**	**Speed (μm/s)**	**Depth (μm)**	**Surface Roughness (nm)**	**References**
wet etching (HF + mask Cr/Au/Cr/Au + SPR220-7)	glass	-	0.78	-	0.24–0.07	300	-	[116]
wet etching (HF 49% + mask Si/Si-carbide/photo-resist)	glass	3000	0.33	-	0.13	1000	-	[66]
HF etching (mask Cr/Au (50 nm/1 μm) + photoresist AZ7220)	Pyrex 7740	±1600	±0.3	44	0.238	500	-	[3]
HF etching (49% HF)	Pyrex 7740	240	0.58	-	0.14	140	-	[117]
HF etching (HFPR-mask)	fused silica	-	0.70	-	0.01	600	10	[118]
DRIE etching (Ni/a-Si/SU-8 masks deep plasma etching)	glass	200	1.25	-	>0.035	250	-	[9]
DRIE (SF_6_ plasma)	Pyrex glass	40–80	>10	10	0.01	200	-	[75]
DRIE (C_4_F_8_/O_2_—Ni mask 8 μm)	glass	3	40	4–14	0.017	120	2–10	[76]
DRIE (SF_6_/Ar—Ni 5 μm)	glass	-	-	-	0.0089	20	1.97	[119]
DRIE (SF_6_—Cr)	glass	-	-	4	0.02	<20	very high	[120]
DRIE (C_4_F_8_/He/O_2_—Si wafer 400 μm)	glass	83.33	3	8–20	0.0083	250	-	[121]
DRIE (C_4_F_8_/He/O_2_—Si wafer 400 μm)	glass	100	3	-	0.0058	300	-	[121]
DRIE (C_4_F_8_/O_2_—Ni mask 5 μm)	glass	22.86	3.5	8–20	0.012	80	-	[9]
DRIE (SF_6_—Ni mask)	glass	20	10	4	0.01	200	4	[75]
DRIE (SF_6_—Ni mask)	glass	-	-	>4	0.0125	40	-	[71]
DRIE (SF_6_/Ar—Ni mask)	glass	-	-	4	0.009	27	-	[71]
DRIE (C_4_F_8_/O_2_—Ni mask 6 μm)	glass	20	6	4–14	0.013	120	2	[122]
DRIE	glass	>1	30	-	0.0055	-	-	[123]
Deep anisotropic dry etching	-	50	-	0.4	0.0001	-	-	[124]
**Thermal Methods**	**Material**	**Diameter (μm)**	**Aspect Ratio**	**Taper Angle * (°)**	**Speed (μm/s)**	**Depth (μm)**	**Surface Roughness (nm)**	**References**
Femtosecond laser (liquid assisted)	-	5–70	40–50	-	30	-	-	[48]
Laser drilling (femtosecond pulses)	fused silica	7–10	3	-	-	30	<HAZ, no cracks, smooth	[52]
Laser drilling (femtosecond pulses)	Foturan glass	56	7.05	-	100–1000	395	-	[55]
Laser drilling femtosecond fiber laser	soda-lime glass	400	2.5	10	-	1000	no cracks, HAZ, rough	[57]
TiSa laser (fs pulse width)	D263T glass foil	208 99	2.4 5.1	15 10	-	500 505	<HAZ, no debris	[53]
Laser (absorbent powder)	glass	200	>12	-	100	2500	-	[58]
Laser drilling (short pulse solid state laser)	Nippon sheet glass	15	0,017	-	-	0.25	no cracks, smooth	[50]
CO_2_ laser	D263T glass foil	<100	>5	3	2000	500	smooth	[13]
CO_2_ laser	glass	71	7,04	3	<2000	500	-	[63]
CO_2_ laser	glass	122	4,10	10	<2000	500	-	[63]
CO_2_ laser (pulsed)	alkali free glass	25	4	-	20,000	100	-	[64]
Laser (CO_2_ infrared laser/Ni grid mask)	-	9.2	0.00043	-	-	0.004	irregular	[43]
LALP (CO_2_ laser, 6W—workpiece immersed in water)	Pyrex 7740	280	1.79	24	11,400	500	no cracks	[62]
Laser (selective etching)	glass	25	40	-	10	-	-	[125]
Focused EDM	Alkali-free EN-A1	20	5	-	500–200	100	-	[4]
	-	65.5	7.6	2	-	500	smooth	[4]
**Hybrid Methods**	**Material**	**Diameter (μm)**	**Aspect Ratio**	**Taper Angle * (°)**	**Speed (μm/s)**	**Depth (μm)**	**Surface Roughness (nm)**	**References**
ECDM	glass	180–40	11	-	1	1200	250–350	[93]
ECDM (pulsed voltage + offset)	glass	455	0.99	-	7.5	450	-	[96]
ECDM (EPDG polishing)	-	210	2.38	0.4°	-	500	5	[88]
SACE (gravity feed)	glass	540	0.37	-	-	200	smooth	[126]
SACE (gravity feed)	glass	600	0.55	-	-	330	HAZ	[126]

***** Taper angle is defined as the angle between the sidewalls of a hole using its cross section (measure of conicity). Consequently, straight holes have low taper angles and conical shaped holes have larger taper angles.
